# PB.38. Extent of agreement between radiological and pathological size and factors affecting completeness of excision in breast-conserving surgery for invasive breast cancer

**DOI:** 10.1186/bcr3744

**Published:** 2014-11-03

**Authors:** C Newlands, M Dixon, L Williams

**Affiliations:** 1 University of Edinburgh/Edinburgh Breast Unit, Edinburgh, UK

## Introduction

Completeness of excision is the most important factor influencing local recurrence for invasive breast cancer. The aim of this study was to determine whether or not radiological underestimation of tumour size increased the risk of incomplete excision in patients undergoing breast-conserving surgery (BCS).

## Methods

This was a retrospective study of 311 women diagnosed with invasive breast cancer and treated with BCS. Data were extracted from patient notes.

## Results

Mean underestimation of tumour size was 11.69 mm for mammography and 17.18 mm for ultrasonography in the incomplete excision group. For every 1 mm increase in mammographical and ultrasonographical underestimation, the risk of incomplete excision rose by 10% and 14% respectively. T stage, maximum tumour diameter, multifocal disease, an *in situ *component and mammographical underestimation all significantly increased the risk of incomplete excision when applied to a best-fit model. The larger the size of the *in situ *component, the greater the risk of both radiological underestimation and incomplete excision.

## Conclusion

Underestimation of tumour size by current radiological techniques increases the risk of incomplete excision in women undergoing BCS. Better preoperative assessment of tumour size is required to reduce this risk. Larger, prospective studies are needed to verify the associations found here.

